# Nutraceutical and Dietary Strategies for Up-Regulating Macroautophagy

**DOI:** 10.3390/ijms23042054

**Published:** 2022-02-12

**Authors:** Mark F. McCarty

**Affiliations:** Catalytic Longevity Foundation, San Diego, CA 92109, USA; markfmccarty@gmail.com

**Keywords:** macroautophagy, autophagy, SIRT1, AMPK, berberine, ferulic acid, melatonin, urolithin A, N1-methylnicotinamide, nicotinamide riboside, glucosamine, ubiquinone, plant-based diets

## Abstract

Macroautophagy is a “cell cleansing” process that rids cells of protein aggregates and damaged organelles that may contribute to disease pathogenesis and the dysfunctions associated with aging. Measures which boost longevity and health span in rodents typically up-regulate macroautophagy, and it has often been suggested that safe strategies which can promote this process in humans may contribute to healthful aging. The kinase ULK1 serves as a trigger for autophagy initiation, and the transcription factors TFEB, FOXO1, ATF4 and CHOP promote expression of a number of proteins which mediate macroautophagy. Nutraceutical or dietary measures which stimulate AMPK, SIRT1, eIF5A, and that diminish the activities of AKT and mTORC1, can be expected to boost the activities of these pro-autophagic factors. The activity of AMPK can be stimulated with the phytochemical berberine. SIRT1 activation may be achieved with a range of agents, including ferulic acid, melatonin, urolithin A, N1-methylnicotinamide, nicotinamide riboside, and glucosamine; correction of ubiquinone deficiency may also be useful in this regard, as may dietary strategies such as time-restricted feeding or intermittent fasting. In the context of an age-related decrease in cellular polyamine levels, provision of exogenous spermidine can boost the hypusination reaction required for the appropriate post-translational modification of eIF5A. Low-protein plant-based diets could be expected to increase ATF4 and CHOP expression, while diminishing IGF-I-mediated activation of AKT and mTORC1. Hence, practical strategies for protecting health by up-regulating macroautophagy may be feasible.

## 1. Boosting Autophagy for Health Protection

Macroautophagy is the process whereby the contents of a cell, including whole organelles, are gradually taken up into forming autophagosomes for degradation in lysosomes, with preference given to the disposal of proteins and organelles that are damaged or marked via ubiquitination. Effective macroautophagy is often referred to as “cell cleansing”, and, when of moderate intensity, and appropriately balanced by synthesis of new proteins and organelles—particularly mitochondria, produced in the complex process of “mitochondrial biogenesis”—it is generally thought to enhance health span [1,2,3,4]. (Henceforth, macroautophagy will be referred to simply as “autophagy”—albeit chaperone-mediated autophagy and microautophagy are distinct processes that also assist in protein disposal within autophagosomes [5,6].) Measures which are known to increase median and maximal lifespan in rodents typically are associated with increased autophagy. Autophagy may be particularly protective with respect to neurodegenerative disorders in which intracellular aggregates of damaged proteins can lead to neuron dysfunction and death [7,8,9]. An age-related decrease in autophagy in lymphocytes appears to be a mediator of the immune senescence that puts the elderly at greater risk for infections and cancer [10]. For these reasons, it is rational to seek safe and practical nutraceutical and dietary measures which can induce a moderate up-regulation of autophagy. An understanding of the molecular biology regulating autophagy is a prerequisite to this search.

## 2. Key Drivers of Autophagy

Activation of the serine/threonine kinase ULK1 is a trigger for initiating the formation of phagophores, which are then elongated into the autophagosomes that engulf cell contents during their formation [11,12]. AMP-activated kinases (AMPK) and mammalian target of rapamycin complex 1 (mTORC1) act as key functional antagonists modulating ULK1 activity [13]. AMPK, which is activated by elevation of AMP+ADP, and hence serves as a monitor for the availability of the biochemically useful free energy provided by ATP, confers an activating phosphorylation on ULK1 [14,15,16,17]. mTORC1, whose activity is enhanced by growth factor activity via AKT and decreased by a cellular deficit of certain amino acids, phosphorylates ULK1 in a way that inhibits its activity [13,18]. The ability of AMPK to suppress mTORC1 activity by complementary phosphorylations of Raptor and TSC2 hence amplifies its positive impact on ULK1 activation [19,20,21].

Like AMPK, the deacetylase SIRT1 functions as a detector of cellular energy deficit; its obligate substrate is NAD+, and a relative paucity of oxidizable substrate enhances its activity by boosting the cytosolic NAD^+^/NADH ratio. SIRT1 activity can stimulate ULK1 indirectly, by increasing AMPK activation via LKB1. The serine/threonine kinase LKB1 activates AMPK via phosphorylation of Thr472 [22]. (Binding of AMP to AMPK aids this phosphorylation, and binding of AMP or ADP impedes its dephosphorylation [14]. Deacetylation of LKB1 by SIRT1 enhances its cytoplasmic localization and its interaction with its activator STRAD1, thereby enhancing its effective activity [23]. Hence, SIRT1 promotes LKB1-mediated activation of AMPK, leading to increased ULK1 activity.

The biosynthesis of autophagosomes and the lysosomes with which they ultimately fuse requires the synthesis of a vast array of proteins. However, a relatively small number of transcription factors can drive the synthesis of most or all of these proteins. In particular, transcription factors EB (TFEB), forkhead box O1 (FOXO1), activating transcription factor 4 (ATF4), and C/EBP homologous protein (CHOP) play a prominent role in this regard, and measures which boost their synthesis and/or activity can thereby enhance autophagy.

## 3. Modulation of TFEB Activity

TFEB is considered the master transcriptional regulator of the synthesis of a great number of proteins required for both lysosome and autophagosome formation [24]. It promotes the transcription of genes carrying a CLEAR regulatory element (coordinated lysosomal expression and regulation) in their promoters, to which TFEB binds; at least 471 such genes have been identified [25]. mTORC1 and ERK2 can phosphorylate TFEB on Ser142 and Ser211; by enabling binding of TFEB to the chaperone 14-3-3, these phosphorylations tend to promote the nuclear export of TFEB, such that it cannot drive transcription [26,27]. Within the cytoplasm, TFEB tends to associate with mTORC1 on the membrane of lysosomes; hence, mTORC1-mediated phosphorylation appears to be the chief mechanism keeping TFEB out of the nucleus [24].

In contrast, AMPK boosts TFEB’s transcriptional activity through several complementary mechanisms. As noted, AMPK functions to decrease mTORC1 activity. Furthermore, AMPK activates the phosphatase calcineurin in the microenvironment of the lysosomal surface, which can reverse the phosphorylations conferred on TFEB by mTORC1 [28,29]. AMPK achieves this indirectly by phosphorylating and activating the kinase PIKFYVE in the lysosomal membrane; this converts phosphatidylinositol-3-phosphate to phosphatidylinositol-3,5-diphosphate [30,31]. The latter can then interact with TRPML1 (also known as MCOLN1) calcium channels to increase their permeability to calcium [32,33]. The resulting increase in free calcium in the lysosomal membrane microenvironment activates calcineurin, enabling it to remove the Ser142 and Ser21 phosphorylations of TFEB [28,29]. Finally, AMPK can itself phosphorylate TFEB; whereas this does not influence TFEB’s subcellular location, it amplifies its transactivational activity [34].

Modulation of TFEB activity by acetylation appears to be complex. On the one hand, up-regulation of this acetylation by treatment with SAHA, an inhibitor of type I, II, and IV histone deacetylases, has been found to enhance TFEB-mediated transcription [35]. On the other hand, SIRT1-mediated deacetylation of K116 in TFEB has been reported to enhance its transcriptional activity by promoting its nuclear import and enhancing its binding to CLEAR elements [36,37]. Moreover, SIRT1 can boost TFEB activity less directly by enhancing AMPK activation. Hence, SIRT1 and AMPK collaborate to induce the nuclear import of TFEB while boosting its transactivational activity.

The synthesis of TFEB is modulated at the translational level by a rather remarkable mechanism. This protein contains two triproline motifs. The efficient translation of such proteins requires optimal activity of eukaryotic initiation factor 5A (eIF5A). This activity, in turn, requires enzymatic activity that converts a specific lysine in eIF5A to the unusual amino acid hypusine; eIF5A is the only mammalian protein known to contain a hypusine residue [38]. This conversion of lysine to hypusine requires the polyamine spermidine as a substrate [39]. Tissue levels of spermidine tend to decline with age, and recent studies show that these levels are sufficiently low in lymphocytes from elderly donors that provision of exogenous spermidine amplifies their hypusinated eIF5A level and thereby boosts the subnormally active autophagy in these lymphocytes [39,40,41].

## 4. Roles of ATF4, CHOP, and FOXO1 in Autophagy

Another transcription factor which promotes synthesis of a number of proteins required for autophagy and lysosomal activity is ATF4. The synthesis of this is enhanced in certain stress situations (the integrated stress response) promoting phosphorylation of eIF2α—a modification which selectively enhances translation of ATF4 mRNA [42]. ATF4, in turn, promotes transcription of the gene encoding the transcription factor CHOP [43]. ATF4 and CHOP collaborate in boosting the transcription of a number of genes coding for autophagosome/lysosomal proteins, whereas the transcription of other sets of genes important for autophagy can be driven by either ATF4 or CHOP acting alone [44]. Genes whose transcription ATF4 and/or CHOP promote include many of those involved in phagophore formation, such as ATGs 3, 5, 7, 10, 12 and 16, as well as Beclin1 [44].

The activity of the transcription factor FOXO1 is modulated by growth factor activity and by SIRT1-reversible acetylation. Growth factor activity, via AKT activation, phosphorylates FOXO1 in such a way that 14-3-3 promotes its nuclear export, abolishing its transcriptional activity [45]. Conversely, a deacetylation of FOXO1 promotes its nuclear import, and also enables it to drive the transcription of the gene coding for the G protein Rab7 [46,47]. The latter plays an essential role in enabling the fusion of late autophagosomes with lysosomes, a crucial step in autosomal flux [48,49].

## 5. Additional Impacts of AMPK and SIRT1 on Autophagy

In addition to up-regulation of ULK1 and TFEB activities, AMPK works in several other ways to promote autophagy [50,51,52]. It confers an activating phosphorylation on the class III phosphatidylinositol-3-kinase PIKC3/VPS34, which plays a crucial role in autophagosome formation [53,54]. Via phosphorylation of FOXO3a on Ser413, it increase the expression and activates the transcriptional activity of this factor, which promotes increased expression of autophagy mediators including LC3B-II, Gabarapl1, and Beclin1 [55,56]. AMPK activity is also required for SIRT1-mediated deacetylation and inactivation of BRD4, a repressor of the transcription of autophagic and lysosomal genes [57].

With respect to SIRT1, its activity also supports autophagy by deacetylating a number of different proteins mediating autophagy, including LC3, ATG5, AT7 and Beclin1 [58,59]. Protein acetylation might be construed as a signal of energy abundance, as the acetyl-CoA employed for protein acetylation derives from oxidation of substrate.

This overview predicts that measures which activate AMPK, SIRT1, and eIF5A, and promote phosphorylation of eIF2α, while down-regulating growth factor activities boosting AKT and mTORC1 activities, could be expected to up-regulate autophagy.

## 6. Nutraceutical Activation of AMPK with Berberine

With respect to AMPK, the nutraceutical berberine, a compound found in a range of herbs used in traditional Chinese medicine, has shown clinically useful activity for control of type 2 diabetes and hyperlipidemia, usually at intakes of 1–2 g daily in divided doses, and these benefits are at least partially attributable to activation of AMPK [60,61,62,63,64]. Indeed, berberine appears to activate AMPK in a manner analogous to the drug metformin, producing a partial inhibition of site I of the mitochondrial electron transport chain; the resulting rise in AMP and ADP enables AMPK to receive an activating phosphorylation from LKB1 [65]. Berberine’s ability to promote autophagy has been demonstrated in cell culture and rodent models [66,67,68].

It should be noted that a number of additional phytochemicals found in medicinal herbs have the potential to activate AMPK. These include compounds found in Chinese medicinal herbs such as mangiferin, astragaloside IV, and triterpenic acids of Cyclocarya paliurus [69]. The traditional anti-diabetic agent bitter melon (Momordica charantia) contains triterpenoids which can activate AMKP via activation of its upstream kinase Ca^+2^/calmodulin-dependent kinase kinase-β (CaMKKβ). Nor should it be forgotten that the prototypical pharmaceutical activator of AMPK, metformin, was developed by chemical modification of guanidine found in Galega officinalis (goat’s rue) [70]. The advantage of berberine is that it is a well-defined chemical compound available as an inexpensive nutraceutical that has an extensive track record of safe and effective clinical use.

## 7. Manifold Nutraceutical and Dietary Options for Activation of SIRT1

The natural lignin resveratrol has been shown to boost Sirt1 activity in rodent and cell culture studies; it is reported to increase Sirt1′s affinity for both NAD+ and acetylated substrate [71]. The basis of its effect in this regard is still unclear; some evidence suggests that it may directly activate Sirt1 via allosteric interaction, whereas other data suggest that its effect is indirect [72,73,74]. In any case, owing to inefficient oral absorption and rapid conjugation (sulfation and glucuronidation) in intestinal mucosa and liver, most efforts to demonstrate that oral resveratrol can activate Sirt1 in human tissues have failed to observe an effect in this regard [75,76]. Efforts to develop resveratrol preparations with higher absorption, and the possibility that resveratrol might exert some Sirt1-independent health benefits, cannot be ruled out [75].

Nonetheless, a number of other nutraceuticals have emerged as potentially useful for SIRT1 activation in recent studies. Berberine can be expected to boost SIRT1 activity, as AMPK, via induction of nicotinamide phosphoribosyltransferase (NAMPT), can increase the availability of SIRT1’s obligate substrate NAD+; NAMPT catalyzes the initial and rate-limiting step in the conversion of free nicotinamide (a product of SIRT1 activity which causes feedback inhibition) to NAD+ [77,78,79]. An alternative way to increase cellular levels of NAD+ is to administer the natural metabolite nutraceutical nicotinamide riboside (NR), which after phosphorylation enters the pathway of NAD+ synthesis. NR has been shown to enhance SIRT1 in a number of cellular and pre-clinical studies, although the dose schedule that might achieve this effect clinically is still being assessed [80,81,82]. When mitochondria are functionally deficient in their obligate cofactor ubiquinone (coenzyme Q10 in humans), the backup of electrons in the electron transport chain which this induces could be expected to decrease the NAD+/NADH ratio, compromising SIRT1 activity; hence, correction of suboptimal ubiquinone status could be expected to aid effective SIRT1 activity [83]. For optimized absorption, ubiquinone is most effectively administered in its reduced form ubiquinol, in nanoliposomes; doses of 200 mg daily have shown clinical efficacy [84,85,86,87,88,89].

Ferulic acid has shown remarkable anti-inflammatory and antioxidant activity in pre-clinical studies and, as sodium ferulate, is widely used in cardiovascular medicine in China [90,91]. Recent studies have found that this may reflect, in large part, the ability of ferulic acid to up-regulate SIRT1 expression at the mRNA and protein level [92,93,94,95,96,97]. This effect has been demonstrated in a number of cell types, and hence appears to be a general effect; its mechanistic basis remains unclear. Dietary anthocyanins, while very poorly absorbed intact, are converted to ferulic acid by gut bacteria, which is then absorbed [98]. It has been postulated that the health benefits attributed to anthocyanin-rich diets may be traceable to ferulic acid [90]. This compound also occurs in conjugated form in a range of whole grains, fruits, vegetables, and legumes; it is partially available from these sources owing to gut esterase activities. In whole grains, ferulic acid serves as a cross-linking agent for arabinoxylan insoluble fiber found in bran; it has been suggested that, in conjunction with phytate, magnesium, and zinc, ferulic acid may be largely responsible for the health benefits associated with regular consumption of whole grains [99]. In a recent controlled clinical study, 1 g ferulic acid daily (500 mg twice daily) was found to decrease C-reactive protein by about one-third in hyperlipidemic patients [100].

Another phytochemical derived from gut bacterial activity is urolithin A, an absorbable metabolite produced from the ellagitannins found in pomegranate and a range of other plant foods; it and its fellow metabolite urolithin B seem likely to mediate the health benefits linked to dietary consumption of pomegranates and pomegranate juice [101,102,103,104]. Several recent rodent and cell culture studies report that urolithin A can modestly increase both the protein expression of SIRT1 and the NAD+/NADH ratio; the basis of these effects remains obscure [105,106]. Urolithin A has only recently become available as a nutraceutical, and further clinical study is needed to establish worthwhile dose levels. Administration of 500–1000 mg daily to elderly humans was found to be safe and associated with increased skeletal muscle markers of mitochondrial biogenesis [107].

The anti-inflammatory and antioxidant effects of melatonin supplementation may be largely attributable to its ability to boost SIRT1 expression at the mRNA level, possibly through activation of the Bmal1 transcription factor, which drives the transcription of genes coding for both SIRT1 and Nrf2 [108,109,110,111,112,113]. Melatonin, usually in doses of 3 mg or more, is commonly administered prior to bedtime, so as not to impede the natural diurnal rhythm induced by this neurohormone.

Whereas nicotinamide can be employed for re-synthesis of NAD+, its catabolism begins with a methylation of its ring nitrogen, yielding the compound N1-methylnicotinamide (MNA) [114]. While initially assumed to be an inactive catabolite, studies nearly two decades ago found that (MNA) has anti-inflammatory activity [115]. Subsequent research has shown that MNA can be protective in rodent models of atherosclerosis, thrombosis, diabetes, metabolic syndrome, polycystic ovary syndrome, Alzheimer’s disease, depression, and age-related hearing loss [116,117,118,119,120,121,122,123,124]. These versatile benefits may be attributable, at least in part, to the ability of MNA to slow proteasomal catabolism of SIRT1, increasing its protein half-life [125]. This effect is associated with decreased phosphorylation of Ser47; phosphorylation at this site by JNK1 prepares SIRT1 for ubiquitination and subsequent proteasomal degradation [126]. How MNA achieves this effect is not yet clear. Evidently, this effect of NMA should be complementary to that of nutraceuticals that boost SIRT1 synthesis and/or enhance the NAD+/NADH ratio. Although MNA has achieved EU approval for use as a nutraceutical, this compound is not yet commercially available as a nutraceutical.

O-GlcNAcylation of SIRT1 has been reported to enhance its deacetylase activity; hence, elevating cellular pools of UDP-N-acetylglucosamine via high-dose glucosamine supplementation may have potential for boosting SIRT1 activity [127,128]. Arguably, this might be pertinent to the modest but significant impact of supplemental glucosamine on longevity in mice, and on the observation that prolonged glucosamine supplementation is associated with decreased global mortality in humans [129,130,131]. The usual dose of glucosamine in management of osteoarthritis is 1.5 g daily, but it has been argued that somewhat higher doses might be required for optimal clinical efficacy; 3 g daily is reported to amplify flow-mediated vasodilation in healthy volunteers—consistent with a role for Sirt1 in boosting endothelial nitric oxide synthase activity [128,132,133].

Calorie restriction and episodic fasting have the potential to increase SIRT1 activity by reducing the availability of oxidizable substrate to cells, and thereby lowering the NAD+/NADH ratio [134,135,136]. These strategies require dedication that may make them impractical for most people, but time-restricted feeding—in which calorie intake is restricted to only one or two meals daily—may be more feasible for many. Eating only in the morning has been shown to boost autophagy prior to resumption of food in the hours prior to food consumption [137]. The senior author has long practiced an alternative strategy wherein aerobic exercise is performed in morning fasting metabolism, and food consumption is postponed to the evening hours; carnitine supplementation might aid this protocol by optimizing the liver’s ability to oxidize fat as a satiety signal [138].

The possibility that ketogenic diets—high-fat diets low in both carbohydrate and protein, such that hepatic ketogenesis is maximized—might influence autophagy and/or Sirt1 activity merits more study. Whereas glucose metabolism via glycolysis reduces cytosolic NAD+ to NADH—an effect expected to diminish Sirt1 activity—ketones are metabolized entirely within mitochondria; moreover, the ATP that this metabolism generates could be expected to suppress glycolysis [139]. Increased markers of autophagy have been observed in the brains of type 1 diabetics who have died of ketoacidotic coma, and increased autophagy has also been observed in rodents fed ketogenic diets or infused with ketones [140,141,142].

Aerobic exercise can enhance AMPK and SIRT1 activity in cardiac muscle and the exercised skeletal muscle by temporarily boosting AMP levels and the cytosolic NAD+/NADH ratio [143,144,145]. Although this activation is transitory, it can exert a longer-term effect owing to activation of transcription factors driving the synthesis of long-lived proteins; hence, both autophagy and mitochondrial biogenesis are promoted.

## 8. Plant-Based Diets May Support Autophagy

A practical way to achieve a modest enhancement in ATF4 production (and hence CHOP as well), while also down-regulating the systemic growth factor activity of IGF-I, may be consumption of a plant-based diet of modest protein content [146]. The enzyme GCN2 is a kinase for eIF2α that is activated by a relative paucity of one or more essential amino acids [147,148]. The ability of GCN2 to activate autophagy hence makes homeostatic sense, as autophagy can increase the availability of essential amino acids for synthesis of essential proteins by degrading existing proteins. Activation of GCN2 in the liver by protein restriction increases hepatic synthesis of fibroblast growth factor-21 (FGF21); ATF4 drives transcription of the gene coding for this hormone [149]. This FGF21 can then act in an autocrine manner on hepatocytes to decrease their responsiveness to growth hormone signaling—an effect which suppresses hepatic secretion of IGF-I [150]. Consumption of vegan or low-protein diets has been associated with decreased plasma IGF-1 and increased plasma FGF21 in epidemiological and clinical studies [146,151,152]. It is notable that genetic overexpression of FGF21 has been found to markedly enhance median and maximal lifespan in mice, and mice with lower IGF-I levels typically experience greater longevity [153,154]. This effect might also help to explain why smaller dog breeds have notably longer lifespans than larger breeds [155,156]. In the middle years of the twentieth century, the traditional Okinawan diet was quite low in protein (9%), and especially animal protein (from just a small amount of fish—4% of calories); the Okinawans were subsequently characterized by having the highest proportion of centenarians in the world [157]. The Okinawans also practiced a modest degree of calorie restriction—an ethos that one should never completely fill one’s stomach, to ensure that enough food is available to all—which also could have enhanced their SIRT1 activity.

## 9. Spermidine for Hypusination of eIF5A

Spermidine, although produced in the body, can also be absorbed from the diet; the richest dietary source appears to be wheat germ. The extent to which post-translational modification of eIF5A to incorporate hypusine (hypusination) can be enhanced by supplemental spermidine needs further study; presumably, this will depend on baseline spermidine status, and might be of greater net benefit in the elderly who may have lesser spermidine tissue stores. Curiously, dietary spermidine content has been shown to correlate inversely with global mortality in a remarkable prospective epidemiological study [158]. Dietary spermidine supplementation conferred cardioprotection in mice subjected to salt-induced hypertension, an effect dependent on autophagy induction [159,160]. Administered in drinking water (5 mM per liter), spermidine has been shown to boost the functional capacity of T and B lymphocytes in aging mice by remedying an age-related reduction in autophagy; if translatable to humans, this effect might improve the natural resistance of the elderly to infections and cancer [39,40,41,161,162]. Spermidine has just recently become available as a nutraceutical (10 mg capsules; typical diets provide about 20 mg daily); the richest common food source appears to be wheat germ [163].

An independent mechanism whereby spermidine may support autophagy is by inhibition of the acetyltransferase EP300, which reduces the activity of certain proteins mediating autophagy via their acetylation [164]. Hence, spermidine complements the activity of SIRT1 in that regard.

## 10. Summary

**Figure 1:**
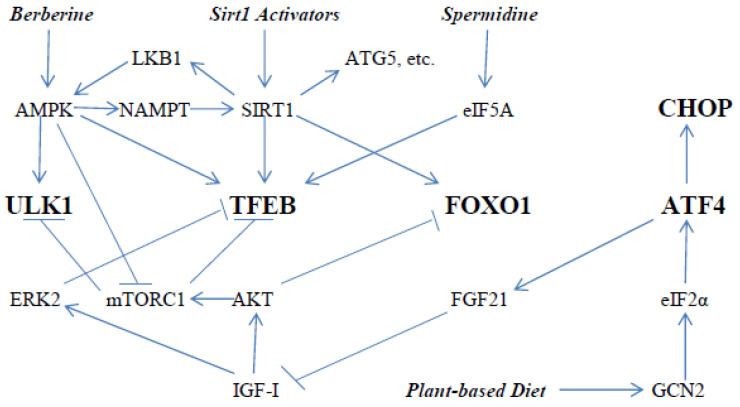
How nutraceuticals and diet can activate key mediators of autophagy—the initiating catalyst of autophagosome formation ULK1, and the transcription factors TFEB, FOXO1, ATF4 and CHOP, which promote expression of many proteins participating in autophagy. Sirt1 can activate some of these proteins (ATG5, LC3, Beclin1, etc.) via deacetylation. Sirt1 activators can include ferulic acid, melatonin, N1-methylnicotinamide, urolithin A, nicotinamide riboside, coenzyme Q10, glucosamine, aerobic exercise, and time-restricted feeding or intermittent fasting.

To summarize, autophagy can be amplified by measures which enhance the activity of the kinase ULK1, and the transcription factors TFEB, FOXO1, ATF4 and CHOP. Measures which stimulate AMPK, SIRT1, and eIF5A, and down-regulate the activity of AKT and mTORC1 (and the growth factors which drive these activities) can be expected to have this effect. Berberine is a clinically useful AMPK activator. A number of nutraceuticals are emerging as of potential utility for boosting SIRT1 activity; these include ferulic acid, melatonin, MNA, urolithin A, NR, and glucosamine (Table 1). Ubiquinol administration may be helpful for this purpose when tissue ubiquinone levels are sub-optimal. Time-restricted feeding regimens may also promote episodic activation of SIRT1. Finally, plant-based diets of modest protein content may promote increased expression of ATF4 and CHOP, while blunting growth factor activity driven by IGF-I. Figure 1 depicts these relationships in graphic form.

It should be noted that activation of AMPK and SIRT1 may exert a number of additional health-protective effects, independent of their impact on autophagy. For one thing, acting primarily through support of PPARγ coactivator-1α activity (PGC-1α), they should promote mitochondrial biogenesis—an effect that is a necessary corrective when mitochondria are degraded by mitophagy [165]. SIRT1 also amplifies the activity of Nrf2 while suppressing the activity of NF-kappaB—accounting for the antioxidant and anti-inflammatory effects of SIRT1 activators [166,167,168]. SIRT1-mediated enhancement of the activities of KLF2 and of endothelial nitric oxide synthase in vascular endothelium can be expected to promote vascular health [169,170,171,172]. In the liver, SIRT1 activity decreases de novo lipogenesis by suppressing the activity of the transcription factor SREBP-1c, while AMPK up-regulates free fatty acid oxidation via inhibition of acetyl-CoA carboxylase—effects that are useful for management of non-alcoholic fatty liver disease [173,174]. Additionally, SIRT1 can lower LDL cholesterol by deacetylating and thereby suppressing hepatic secretion of PCSK9—a protease that binds to the external surface of hepatic LDL receptors and routes them to lysosomal degradation [175,176]. Although SIRT1’s ability to lessen p53 activation prompted by DNA damage has the potential to aid cancer promotion, this is compensated by SIRT’s ability to promote DNA repair, so that the net impact of SIRT1 activation on cancer risk remains equivocal [177].

With respect to the ability of low-protein plant-based diets to activate the GCN2-eIF2a-ATF4 pathway, this pathway, which enhances expression of FGF21 and adiponectin, may help to rationalize the lower risks for obesity, diabetes, cardiovascular disorders, autoimmunity and some “Western” types of cancer that have characterized some quasi-vegan societies [146,178].

Finally, it should be noted that, whereas moderate up-regulation of autophagy is often health protective, excessive autophagy, when not matched by compensatory anabolism, can lead to autophagic cell death [179,180,181]. Whereas up-regulation of autophagy appears to be beneficial in the early stages of various neurodegenerative diseases entailing accumulation of pathogenic protein complexes, it may be counterproductive in later-stage disease [182]. Additionally, some cancers employ elevated autophagy to enable them to survive in microenvironments low in oxygen and nutrients—pancreatic cancer is notable in this regard [183]. Hence, whether boosting autophagy is clinically worthwhile may hinge on the clinical context.

## 11. Dedication

This essay is dedicated to the memory of Jeremy Stone, a close friend of mine who was fascinated by the potential of up-regulated autophagy for promoting healthful longevity, and who encouraged me to pursue this topic. Jeremy, the eldest son of the great progressive journalist Izzy Stone, was President of the Federation of American Scientists for 30 years, under whose aegis he undertook private diplomacy throughout the world, aimed at preventing war and nuclear catastrophe. Perhaps the most celebrated of his many achievements was to conceive and enable the implementation of the Anti-Ballistic Missile Treaty. Jeremy, this one is for you!

## Figures and Tables

**Table 1:** Nutraceuticals with potential for activation of autophagy.

Nutraceutical	Target	Clinically Relevant Dosing *	References
Berberine	AMPK	1000–2000 mg daily, divided doses	[60,61,62,63,64,65,66,67,68]
Ferulic Acid	Sirt1	500–1000 mg daily, divided doses	[85,86,87,88,89,90,91,92]
Melatonin	Sirt1	5–20 mg at bedtime	[104,105,106,107,108,109]
N1-methylnicotinamide	Sirt1	Not established **	[121]
Urolithin A	Sirt1	Not established **	[101,102,103]
Nicotinamide Riboside	Sirt1	500–2000 mg daily, divided doses	[75,76,77]
Coenzyme Q10	Sirt1	100–300 mg as ubiquinol daily	[78]
Glucosamine	Sirt1	1.5–3 g once daily	[123,124]
Spermidine	EIF5A ***	10–30 mg daily, divided doses ****	[39,40,41]

* These are not represented as ideal doses for activation of the defined targets, but rather doses which in clinical experience have been sufficient to exert physiological effects. ** While these agents are produced in the human body and can be presumed to be safe at physiological levels, and have been made available as nutraceuticals, there is too little published clinical experience with them at present to define worthwhile clinical doses. *** Required for efficient translation of TFEB mRNA. **** Since natural diets provide a spermidine intake in the range of 20 mg daily, it is presumed that 10–30 mg of supplemental spermidine daily will meaningfully impact spermidine status.

## Data Availability

Not applicable.
